# Co-producing access to care with young people living in Zambia through digital and vending machine-enabled innovation in sexual and reproductive health: A place-based, human-centred design research approach

**DOI:** 10.1371/journal.pone.0356398

**Published:** 2026-08-27

**Authors:** Mary Darking, Helen Ayles, Steve Belemu, Paloma Chausa, Francisco J. Gárate, Enrique J. Gómez, Melleh Gondwe, David Lane, Deborah Kaluba-Milimo, Monde Mwamba, Musonda Simwinga, Jennifer Whetham, Jaime H. Vera

**Affiliations:** 1 College of Creativity, Technology and Society, University of Brighton, Brighton, United Kingdom; 2 Department of Clinical Research, London School of Hygiene and Tropical Medicine, London, United Kingdom; 3 Zambart Research, Lusaka, Zambia; 4 Biomedical Engineering and Telemedicine Centre, ETSI Telecomunicación, Center for Biomedical Technology, Universidad Politécnica de Madrid, Madrid, Spain; 5 EmERGE mHealth Ltd., Chichester, United Kingdom; 6 Lawson Unit, University Hospitals Sussex NHS Foundation Trust, Brighton, United Kingdom; 7 Department of Global Health and Infection, Brighton and Sussex Medical School; University of Sussex, Brighton, United Kingdom; University of Oxford Nuffield Department of Clinical Medicine: University of Oxford Nuffield Department of Medicine, UNITED KINGDOM OF GREAT BRITAIN AND NORTHERN IRELAND

## Abstract

Digital health innovation can fail to move beyond prototype or pilot stages or achieve intended outcomes due to a lack of involvement and attunement to local context. This article shares the potential of place-based co-production to support the development, acceptability and usability of context-attuned, digital health service innovation for young people. To illustrate the approach, we report on a collaborative, interdisciplinary project involving a Zambian public health research partner based in Lusaka, Zambia, three European universities and a not-for-profit digital innovation partner. Our youth-led, co-production process involved 6 stages: recruitment of youth Co-design Leaders; role play-based care pathway co-design; app co-design; digital prototype development; stakeholder consultation; and digital pathway testing. We used a combination of qualitative social science research, community and arts-based, human-centred design and digital innovation methods. Young people traced the digital testing and sampling process from accessing self-testing and sampling kits to laboratory processing of results through to how results should be communicated. They were encouraged to evaluate how these processes would work in the context of their everyday lives. We systematically embedded this ideation work into a technically functional ‘eHealth Yabwela’ app and clinical or laboratory worker-facing web application which young people user tested. Our place-based focus encouraged elicitation of whole pathway, *in situ,* logistical feasibility alongside social and technical concerns, enabling us to engage with potential adoption and accessibility barriers during the design process. Youth-led co-production created the conditions for participants to lead discussions, voice requirements and hold the project team accountable. Young people recognised that advocacy, overcoming digital inequalities, and further co-production with healthcare workers already facilitating youth-friendly healthcare provision would be needed to integrate the pathway within local systems.

## 1. Introduction

Globally, uptake of HIV and STI testing among young people in conventional clinical settings remains suboptimal [1]. This is an issue because knowing HIV status contributes to prevention of onwards transmission and can be a first step in accessing treatment [2]. Curable sexually transmitted infections (STIs) can cause serious morbidity, including pregnancy complications and infertility, and are well-recognised risk factors for HIV transmission [3]. There is also strong synergy between STIs and HIV, especially in young people, in whom high rates of untreated STIs may help explain high HIV incidence [4]. Women and girls are disproportionately affected by these issues, which along with lack of access to birth control, can hold consequences for their social and economic wellbeing across the lifecourse [5,6]. In 2022 the World Health Organization (WHO) described access to sexual health services for adolescents as “lagging behind the progress achieved for adults” [1]. Young people in sub-Saharan Africa experience social, economic and political barriers to accessing mainstream sexual and reproductive health (SRH) services [7]. Many say they feel judged or else are discriminated against when they attend health facilities seeking support with STIs, HIV testing and contraception [8–10]. On this basis, global health policy guidelines call for integrated, person-centred responses that simultaneously address stigma and discrimination [11].

HIV self-testing and STI self-sampling are recognised by policy makers as having the potential to overcome some of these barriers [12]. However, their distribution and uptake in sub-Saharan countries such as Zambia has proved challenging [13,14] and until recently there has been limited public health, laboratory infrastructure to support aetiological diagnosis of common, curable STIs such as chlamydia, gonorrhoea and trichomoniasis. Recent changes in testing capability in the form of portable, “container laboratories” comprehensively equipped with laboratory testing equipment and computational technologies offer new opportunities for innovation [15]. Translating the value of these new capabilities into improved, accessible sexual and reproductive care for young people was the key challenge this research sought to address.

On this basis the eHealth Yabwela project co-produced an integrated health service design and prototype technologies aimed at expanding access to HIV self-testing, STI self-sampling and aetiological diagnosis for young people (age 19–23) living in Lusaka, Zambia. From an intervention design perspective, even with testing infrastructure in place and self-testing and sampling kits available, creating a locally attainable pathway of care that young people feel confident accessing is a complex question with no “globally generalisable” [7] solution. Building on local evidence of acceptability and adapting successful examples of service implementation to local contexts is therefore imperative. In Zambia, previous youth-focused service innovation has included: the creation of peer-led, neighbourhood-based community hubs [16]; the development of youth-friendly spaces at health facilities [17]; and the use of digital vending machines that can dispense SRH products from different locations [18]. These intervention designs were found acceptable to young people particularly in the early stages of seeking help and support with SRH issues. Alongside these research programs, long term relationships with local communities, young people, healthcare professionals and stakeholders have been built [19] and a high level of methodological expertise [20,21] acquired in the meaningful engagement of young people [22] and need for non-Eurocentric approaches [23].

We combined this track record of research on access to SRH services in Zambia with co-designed mHealth research and innovation on improving access to HIV care (the EmERGE project and pathway) [24]. This research showed that use of mobile phones in the delivery of health services, or mHealth, can support people-centred improvement to care and access to health information that results in a reduced number of visits to health facilities and reduced expense to patients, whilst maintaining connection to care, and health outcomes [25,26]. The EmERGE pathway was successfully implemented in five clinics, proved sustainable post-project at two clinics and subsequently saw its technical architecture (a “design framework for organizing IT capabilities” [27]) re-purposed for use in HIV prevention. Building on this evidence base, this project sought to establish if combining these components (digital vending machines; mobile phones; HIV self-testing and STI self-sampling kits; and developments in laboratory infrastructure) to create an integrated person-centred experience of SRH care and a health care pathway was feasible, acceptable and usable in pilot form to young people living in Lusaka.

Over the course of 18 months the research and innovation reported on here sought to 1) co-produce a fully-specified service design for this pathway with young people 2) co-design functional prototype technologies and 3) test and assess the feasibility and acceptability of the pathway with young people and local stakeholders. To achieve this, we combined youth-led co-production, place-based human-centred design and community-based participatory research methods into a phased research design fully integrated within the digital innovation process.

Our youth-led, interdisciplinary project team (based in Zambia, UK and Europe) co-produced a technically functional, vending machine and app-enabled care pathway and user journey. Our Zambian youth leads together with the study research staff chose the name ‘eHealth Yabwela’ for this project. Yabwela means ‘it has come’ or ‘it is has arrived’ in Nyanja (Chewa). In the context of eHealth Yabwela, the name can be interpreted as “eHealth has arrived” or “eHealth is here” conveying the message that digital health services are now available and accessible. Nyanja is one of Zambia’s most widely spoken languages and a language commonly spoken in Lusaka along with Bemba [28].

We argue that designing integrated digital health services aimed at addressing inequity of access requires co-produced, people-centred, place-based innovation approaches. Population level health data, even when focused on key populations, can fail to elicit complex lived experience of SRH need. Furthermore, health access equity is often context specific and can be hyperlocal in ways that public health or service level data cannot capture. This holds practical implications for how relationships between service uptake and health inequity are known and acted on. On this basis, we argue there is a distinct need for research and innovation methods capable of i.) recognising under-represented experience ethically and empathetically and ii.) embedding needs arising from those experiences into service design transparently and accountably. In what follows, we demonstrate how place-based, human-centred design and youth-led co-production informed our approach to: i) pathway and technology co-design, ii) prototype development and testing and iii.) deliberation on acceptability, viability and implementation requirements.

## 2. Theoretical and conceptual framework

The theory of Sociotechnical Transformation used in this research has been widely recognised as an effective approach to the qualitative study of complex, digital innovation [29]. Developed within the field of Information Systems, it is informed by the anthropological work of Tim Ingold [30,31]. Focus is placed on “flows” (or processes and inter-relationships) and their *in situ* making [32,33]. Social scientists take specific interest in the “conditions of possibility” [34] that shape how processes are bounded, who or what these boundary-making practices exclude and what the social, material and historical contexts of those exclusions are [35,36]. In the context of healthcare, this is particularly relevant where intervention design calls for different types of knowledge to be mobilised, yet not all knowledge types have equal status [37].

Encompassing similar concerns, the underlying principles of co-production have a long history in public services [38,39], service user movements [40,41] and participatory research design [42,43], but as a term, has only recently been used in health policy and practice [44,45]. In the UK, co-production has been defined as an equal partnership which engages groups of people at the earliest stages of service design, development and evaluation [46]. Focus is placed on engaging people who have been historically marginalised, under-served or excluded from health systems. In this context, co-production signals a strong commitment to the pro-active co-creation of equal voice and shared decision-making. It calls for collaboration in practices ranging from how activity is governed, managed and led, to how conditions for equal voice are created [47]. Co-production therefore combines what Hayes et al. [34] refer to as front stage (interactionist) and backstage (transformational) participatory approaches whereby the latter aims to support deeper, long term, socio-political and system change [48].

Here we use the term place-based co-production to argue that research and co-design methods that draw people’s current lived experience of communities, places and systems to the foreground of decision-making make an important contribution to the design of locally viable and acceptable health services and interventions [49,50]. Such methods provide a basis for taking action in situations where there is tangible need but i.) local, population-level data is partial, incomplete or out of date, ii.) lived experience is complex and under-reported and/or iii.) the people for whom equity of healthcare access are sought wish to avoid engagement with services. They also enable knowledge of local systems, services and infrastructure to come to the foreground through frontline, stakeholder and lived experience dialogue. This is significant because integrated health care intervention design requires the combination and recombination of existing spaces, settings and services and partnership working with local stakeholders who share an interest in meeting local health equity needs. Here, we demonstrate that eliciting this knowledge is supported by collective, *in situ* making and deliberation with the people for whom system improvement is sought. The concept of *place* has long been central to social and geographic research because it supports forms of inductive, abductive and mixed methods inquiry that are attentive to lived experience, institutional relations and spatial variation [51,52]. In this study, a place-based approach directed evidence gathering toward questions of equity, viability, and acceptability as these emerged through discussion and practical engagement in a co-design process. From this starting point, we undertook a youth-led co-production process to develop a digitally enabled approach to youth engagement with SRH care in Lusaka, Zambia.

## 3. Research design

This article brings together research results and digital innovation outputs from two previously distinct programs of research. The first program is the co-design, development, implementation, evaluation and commercialisation of mobile phone-based, digital health pathways in HIV undertaken by the EmERGE consortium in five European countries 2015−20 [24]; subsequently re-purposed for HIV prevention (UK only) 2020−24 [53]. The second program is the co-design, development, implementation and evaluation of digital vending machines undertaken in the UK [54] and Zambia [18].

The project reported on here was developed in partnership with Zambart a public health research organisation based in Lusaka, Zambia. Zambart social science and community engagement co-researchers recruited 12 youth Co-design Leaders over the course of the project and with them coordinated the development of: Phase One service design and technology specification; and Phase Two user testing and stakeholder engagement workshops. They were supported by a UK social scientist specialising in digital innovation, participatory human-centred design and co-production. Data collection and analysis were undertaken by this team. Clinical input was provided by three team members who practice as senior medical doctors in the field of HIV, Sexual Health and Infectious Diseases. Leadership in technology development was provided by Universidad Politecnica de Madrid and not-for-profit digital innovation partner EmERGE mHealth Ltd. All participants in each group (young people, service providers and community stakeholders) were over 18 and provided informed, written consent for every phase of research in which they took part. Co-production leads provided informed, written consent for the duration of the project as part of which they opted to forego anonymity and gave permission for photos and videos of them and the groupwork they engaged in to be shared publicly.

**Fig 1:**
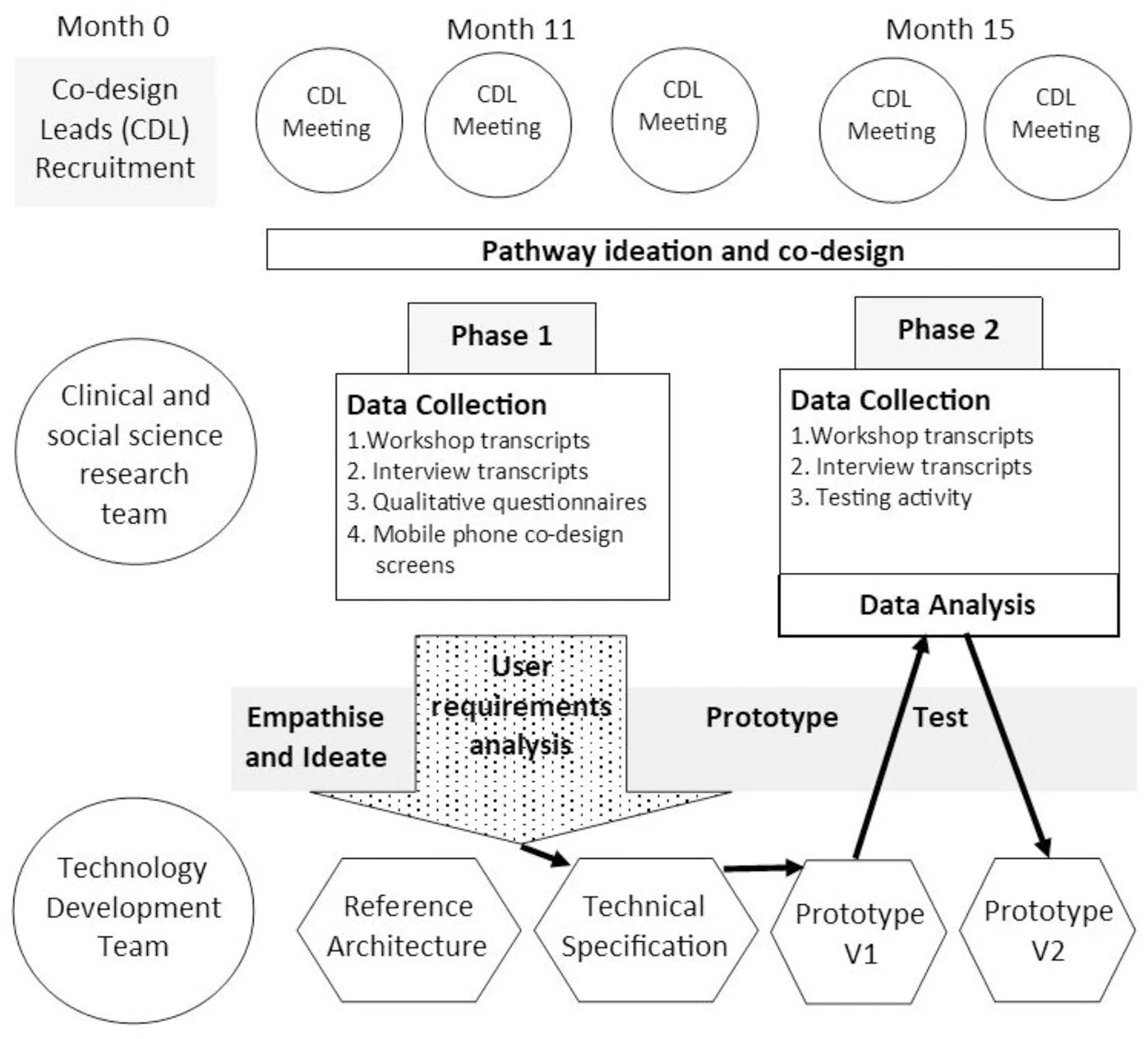
Integrated research design for human-centred, digital health innovation co-production.

Our research objectives required integration of four distinct processes: 1) a youth-led, co-production process; 2) a digital health service innovation process (concept only); 3) a technology development process (functional technical prototype only); and 4) a qualitative, social science research process. Youth ‘Co-design Leaders’ (CDL) worked with us in equal partnership over a 15-month period engaging in training (research methods and ethics), project meetings, regular updates and workshops (represented as CDL Meetings in Fig 1 below). As co-production progressed, a human-centred design (HCD) cycle [55,56] was introduced which formed a bridge between co-design activities undertaken with young people and the technology development process. The HCD process, drawn from design science, followed a conventional ‘empathise; define; ideate; prioritise; prototype and test’ cycle [57,58]. A multi-phase, qualitative, socio-technical research design was integrated within co-production and innovation activity via a set of ‘co-design workshops’ held in each phase which included multiple, qualitative data collection and design methods.

Recruitment of Co-design Leaders (n = 12) and recruitment of participants for Co-design workshops (n = 44) was based on the same criteria for inclusion and exclusion. These criteria were informed by our place-based, health equity focus, attentiveness to digital inequality and the sensitivity of SRH conversations. Young people aged 19–23 were recruited and it was required that at least half of workshop participants should be women due to the additional barriers to education and engagement in Science, Technology, Engineering and Maths (STEM) subjects they face. It was required that participants should live in Lusaka but come from a range of different local neighbourhoods. The neighbourhoods selected included those with high-density housing recognised as low-income neighbourhoods and some medium and low-density neighbourhoods. Participants came from a range of educational settings including secondary schools, colleges and universities. Chipata was chosen as a focus-neighbourhood in order to build on knowledge and relationships developed through previous projects and so this neighbourhood was over-represented compared to others. In addition, recruitment information asked that potential participants should i) own or have access to a smartphone and have used smartphone apps and 2) be interested, able and willing to engage in challenging learning activity. By creating selection criteria that included a range of neighbourhoods and settings we attempted to mitigate the risk that requirements for familiarity with smartphones would unintentionally exclude young people coming from different socio-economic backgrounds from taking part in the research.

Over the two phases 56 young people participated as Co-Design Leads (n = 12) and as co-design workshop participants in Phase One (n = 20) and Phase Two (n = 24). In addition, focus groups were conducted with community stakeholders from Chipata Compound (11), policy maker stakeholders (7) and healthcare providers (9) based in Lusaka.

### 3.1 Materials and methods

Ethical approvals were granted by the University of Zambia Biomedical Research Ethics Committee (UNZABREC REF. NO. 3435−2022). Co-design Leads met nine times over the course of the project with an average of eight Leads attending each meeting. In month 11, they and the project team co-designed the format of Phase One workshops (repeated a further three times; n = 20 participants) and in month 15 they co-designed the format of Phase Two workshops (repeated three times n = 24 participants). Data collection took place in workshops with young people (four workshops in Phase One; four workshops in Phase Two) or else in stakeholder/provider focus groups which were recorded and transcribed. Recruitment took place between 16/01/2023 and 24/11/2023. Prior to each research phase, youth Co-design Leads were supported to design and pilot each workshop. Where possible one or two co-design leads attended each subsequent workshop to represent the other leads, support delivery and ensure fidelity to their concepts and ideas. Arts-based methods [59,60] including – singing and dancing, collage making, installation building, role play and video-making – were co-created with youth Co-design Leads, community engagement leads and the social science team to support young people’s engagement, voice building and ideation. These were informed by social design methods that seek to materialise data and digital infrastructure to support collective debate and deliberation [61]. Images of two large mobile phone screens and sticky notes were used in Phase One workshops to develop ideation around data privacy, user requirements and functionalities with photos of these screens submitted to data analysis.

As a multi-method research design that aimed to produce and test functional prototype technologies in a defined time period, the analytical strategy adopted was sequential, pragmatic and composite. Workshops were analysed using a democratic-pluralism [62], social design approach. This involved collating ideas in real time through transparent, consensus-based dialogue. All ideas voiced were valued, deliberated on and where relevant problematised. The process supported decision-making and sharing of outcomes across workshops and phases, while still allowing space for active challenge and debate (“agonism” [62]) within the process. Arts-based methods led to the co-production of installations and exploration of their sequence and positioning through role-play based, physical enactment of the pathway (see Fig 1). In the app design process, a simple form of qualitative content analysis was formulated to further embed principles of transparency and democratic-pluralism. All ideas were retained including those that contradicted each other and one-off ideas. Where indicated, strong sentiment and clear consensus were noted. Collated ideas were passed onto the technical team who undertook a prioritisation exercise based on time, budget and available expertise aligned to Phase Two functional prototype testing activities planned for three months later. This was shared back with Co-design Leads in an iterative process that concluded with their sign-off on proposed developments. At the end of the process, the results of youth-led work were shared with local community stakeholders and healthcare workers. Here again, points of consensus and points of difference between youth and other stakeholder voices were noted. Whilst all workshops were recorded, in-depth, qualitative analysis of FGD and workshop transcripts was not undertaken in order to direct appropriate time and resource toward innovation outputs.

Below, we share findings on the innovation process co-produced through our research and demonstrate how the theory of Sociotechnical Transformation [30] and approach to data analysis enabled us to embed young people’s voices and place-based priorities into the technical specification and technically functional prototypes.

## 4. Findings

### 4.1 Co-designing the eHealth Yabwela pathway: Place-based engagement, whole pathway ideation and role play

Phase One workshops were co-designed with our youth Co-design Leaders and divided into two parts. The first part co-created and explored the access to healthcare journey from a position of empathy. Participants were encouraged to locate and ground their contributions in their current, expert, lived experience; to think ‘as a young person living in Lusaka now’. In this way, youth leaders and co-designers were recognised as uniquely positioned to engage in problem identification and develop problem-solving logics through which we would find out collectively if the proposed technologies would work.

For this part, potential pathway components were discussed, materially represented and subsequently set out around the workshop space by the youth Co-design Leads and research team. Installations were created at different points around the room (referred to as ‘stations’ by participants). They created six installations which they could visit, discuss and materially engage with in order to support their ideation of the journey they were creating. Once the components were established, in subsequent workshops, each installation was covered and only revealed once participant discussion instinctively progressed toward wanting to explore what ‘the next step’ would be. At times, this order closely reflected a practical sequence of activity such as: 1) collecting 2) using a self-testing or sampling kit and 3) receiving results from laboratory. At other times the sequence was less clear or else required opening out and exploring. In the example above, for instance, questions of *where* a young person could engage comfortably in self-testing and sampling, knowledge of how to use the kits and practical questions of *how* they would dispose of wrappers (confidentially) were raised.

The focal scenario and question around which Phase One workshop activities were organised were: “a young person living in Lusaka needs support to access the care they need. How could a mobile phone and vending machine help?” (Workshop Facilitator prompt). In the workshop space the digital vending machine was represented by a life-size banner, printed with an image of a machine that had been piloted in Lusaka, along with a short video of the machine being used. Vending machines are not common in Lusaka so most participants had not seen one. However, there was shared experience of seeing vending machines in films or TV series, with the most cited example being a snack vending machine located in a school or hospital waiting room. Attentiveness to place supported discussions of where vending machines could be located which in turn elicited discussion of the barriers to access they could help to overcome (including difficult experiences of using local pharmacies; and finding, traveling to and waiting in queues at health-facilities). Hyperlocal discussions of ‘where’ machines could be located highlighted the need for sensitivity to community, confidentiality and security, prompting comparison with Automatic Teller Machines (ATMs), which are common in Lusaka. Shopping malls were identified by co-designers (and community stakeholders) as viable locations, normalising access and affording an element of anonymity by reducing potential for a young person to be recognised in their immediate community.

Having been recruited on the basis that they owned or had access to a smartphone, participants engaged in peer-based discussions of mobile phone models, data use, costs, prevalence of ‘small phones’ (non-smartphones) compared to smartphones and first experiences of seeing, borrowing and having their own phone, stimulating ideation. As their ideas took form and the potential benefits of the pathway became clearer, youth co-designers began to problematise the technology-centred, city-focused characteristics of the proposed model. They began to deliberate on differences in infrastructure and communication needs in places within and outside the city which motivated discussion on questions equity. Co-designers primary concern was that a pathway that depended on smartphone ownership would compound existing socio-economic, digital and healthcare access inequity. They discussed how people living in high density housing neighbourhoods may lack access to infrastructure, be subject to long electricity and mobile network outages, or lack money to buy data. For these people, a smartphone-dependent approach to accessing healthcare could compound existing experience of inequity. Designing ways in which access inequity could be overcome became a primary and non-negotiable focus for young people who advocated energetically on these points. Once highlighted, workshop focus would invariably shift toward ideating variations of the pathway that included a non-smartphone (or “small phone”) pathway using USSD technology and an “outreach pathway” where no mobile phone or vending machine was needed at all (just the web application, sampling kits and a healthcare worker operated portable device). Whilst there was insufficient time to produce functional prototypes of the technologies needed to test these pathways, background research on USSD-based SRH innovation was conducted by the technical team [63] and ‘on paper’ technical specifications developed to enable future prototyping and implementation.

In addition, participants sought ways to mitigate inequities of access by co-designing smartphone app and vending machine capabilities to address communication and accessibility needs. Youth leaders and co-designers educated one of their (European) workshop facilitators on the significance of local languages in Zambia: their number (72) and variations; their history and ties to places and regions; their relationship to tribal and family ties; and to people’s first and family names. They also highlighted that whilst Zambia retained English as its official language for the purposes of state administration and education, local languages play fundamentally important social and cultural roles. Therefore, ensuring the vending machine and app had options that enabled their user interfaces to appear in English and at least two local languages was non-negotiable. For this reason, functionality to translate interfaces into Bemba and Nyanja (as a starting point) was designed into functional prototypes. Feedback from app prototype testing in Phase Two highlighted that young people had never seen a piece of technology, particularly a smartphone application, where textual components of the interface appeared in a local language.

**Fig 2:**
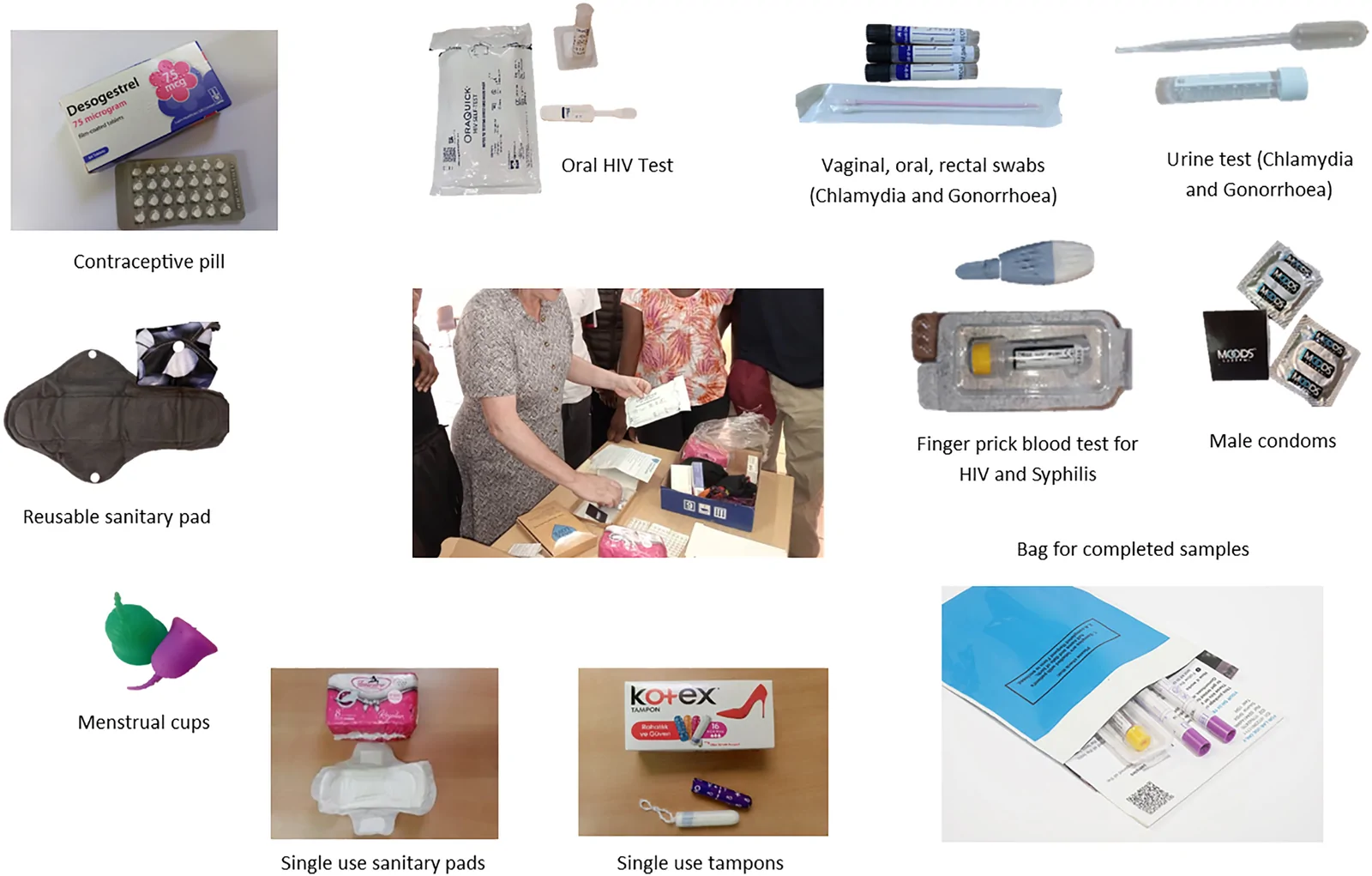
Exploring sexual and reproductive health products.

Using materialisation and role play techniques co-designers were able to move freely between physical, digital, embodied and data-focused aspects of the pathway. For example, one of the installations created was a table with various local SRH products on it along with testing and sampling kits which included swabs and finger prick HIV tests (Fig 2). Young people were encouraged to think about their own experience and those of friends and peers who might seek support with SRH concerns and ask whatever questions occurred to them. They were encouraged to open up the testing and sampling kit boxes and packets as well as pouches containing menstrual cups and reusable sanitary towels. Particular focus was given to the STI self-sampling kits as these interventions had not been available previously.

After this, discussions turned to where samples should be deposited, how they would be collected and where they would be taken. In the UK, samples (placed within a ziplock biohazard bag and freepost box) are returned to the local hospital via the national postal service and then transported to a contracted laboratory [64,65]. In Zambia, participants recognised that a place-specific collection and delivery system would be needed. To explore these questions, Co-design Leaders introduced a cardboard ‘deposit box’ and created the role of ‘driver’. Public health facilities in Lusaka depend on STI diagnosis based on presenting symptoms (syndromic diagnosis) rather than aetiological diagnosis (except in the case of pre-natal care and syphilis). Therefore, samples would need to be returned directly to a public health laboratory with sample processing capacity. The role of ‘laboratory worker’ was therefore created and a laboratory gown, goggles and gloves were placed on one of the tables. Workshop participant volunteers took the roles of ‘driver’ and ‘laboratory worker’ respectively, claiming the appropriate clothes and props for their role.

At this point, use was made of the fact that there were container laboratory facilities in the grounds of Zambart, the public health research organisation where workshops took place. Laboratory staff were approached and asked if workshop participants could physically visit the laboratory and with their agreement, workshop participants led by the volunteer lab worker and driver, carried the deposit box to a serology and immunology lab. They were greeted by a laboratory IT worker who continued the role play, acting out exactly what processes they follow when they receive samples from drivers who arrive at the laboratory. She showed them the workflow involved in processing samples to the point where test results are sent from laboratory machines to a computer based in the lab and then to local health facilities. Here, the suggestion was introduced: ‘what if’ a software application was installed on the laboratory computer to send test results to an individual’s mobile phone as well as to the health facility? On this basis, participants were asked to imagine what kind of smartphone application design would support young people to receive, understand and act on test results. This design activity formed the focus of the second half of Phase One workshops.

**Fig 3:**
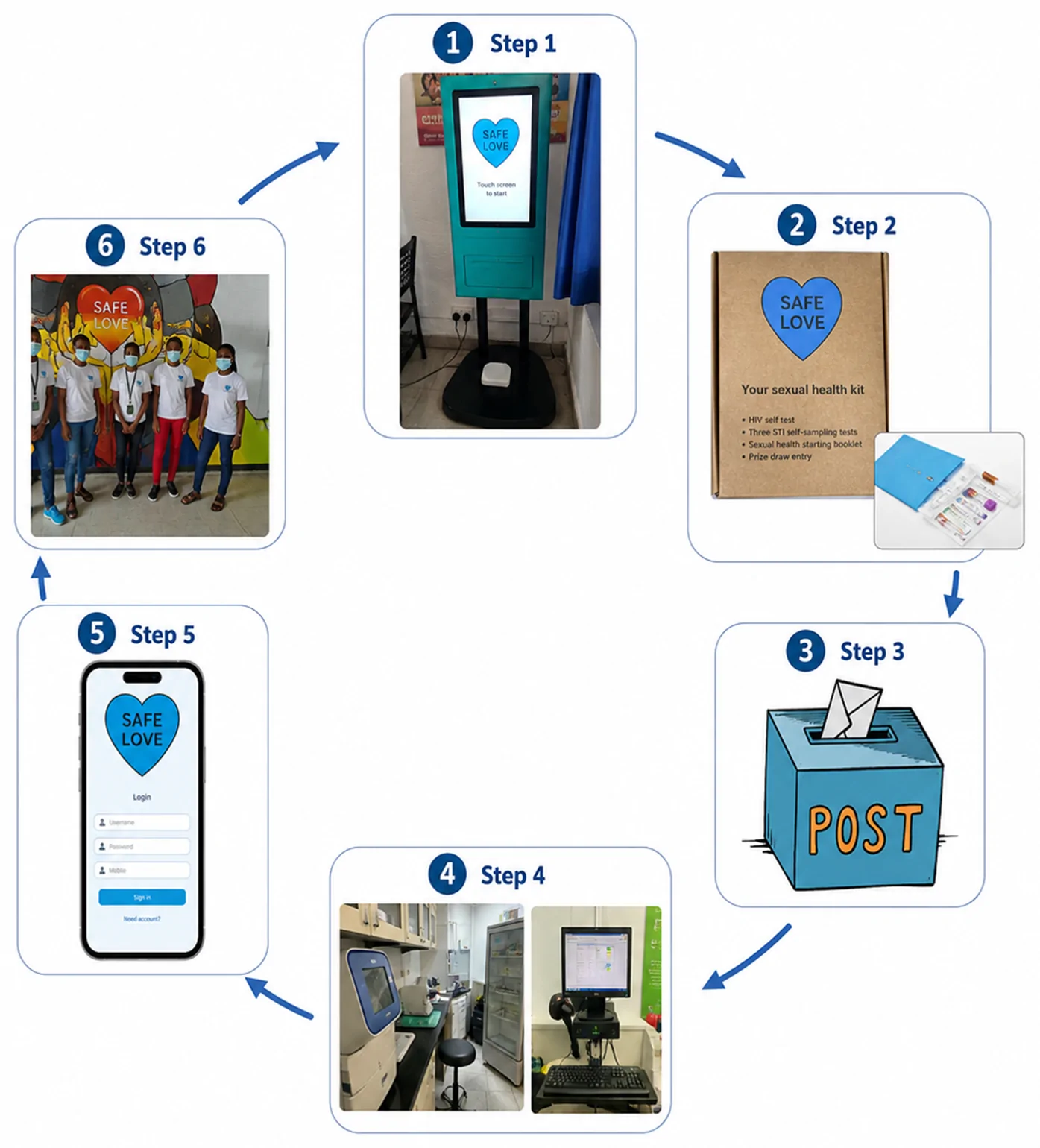
The eHealth Yabwela pathway.

From the first Co-design Leaders workshop and subsequently over the course of 3 further workshops with youth co-designers, the lived experience of a young person seeking help with SRH needs was interwoven with the ‘eHealth Yabwela pathway’. Designing through discovery and making the pathway together had been highly valued by Co-design Leaders and they suggested that adopting the same inquiry-based mode would engage other young people. For the wider research team this suggestion was valuable because it enabled them to test if the logic and sequence of activities identified in the original workshop (with eight youth Co-design Leads) presented similarly or differently with participants in subsequent Phase One (n = 20) and Phase Two workshops (n = 24). By these means, the eHealth Yabwela journey or pathway (Fig 3) was defined.

Whilst more and different questions arose and the need for more detailed specification grew, successive workshops confirmed that the pathway and proposed ‘steps’ were considered: viable (can this be made to work here in Lusaka?); acceptable (would *you* use this pathway?); and usable (is the pathway easy to use and learn about?) to 100% of young people attending co-design workshops (though on usability there were clear requests for supportive instructions and educational resources).

### 4.2 ehealth Yabwela software co-design: Technical specification and development

The second part of Phase One workshops followed directly from immersive, pathway co-design activities and involved the same participants. Its focus was the co-design of the smartphone application that would enable and support pathway use.

Our research project aims required us to create technically functional prototypes of the technologies needed to produce the eHealth Yabwela pathway. A potential technical architecture (known as a reference architecture) for the digital and data elements of the pathway had been proposed at the research design stage. This proposal was based on evidence from the EmERGE project which successfully co-designed, developed and implemented a digital platform for HIV care in five European clinical sites (2015–2020), each with highly varied and distinct technical capabilities and data environments. Despite this, research team members who had been involved in the EmERGE project knew that without *in situ* exploration of what technologies could be locally supported and what data were available, it was not possible to confirm the viability of the technical architecture in advance. In previous research, the technical architecture included: a service user-facing smartphone app; a laboratory or clinic-facing web application; and software relevant to messaging and interfacing or integration (to enable data to move between digital components).

From a digital standpoint, the technical components that were open to participant design or re-design were the smartphone application and the laboratory or clinic-facing web application. Elements of the integration software, or Application Programme Interfaces (APIs), would also require (re)building. However, the requirements for this would stem directly from what digital infrastructure and data elements were a) available in this context and b) identified by co-design participants as important for the app to display.

**Fig 4:**
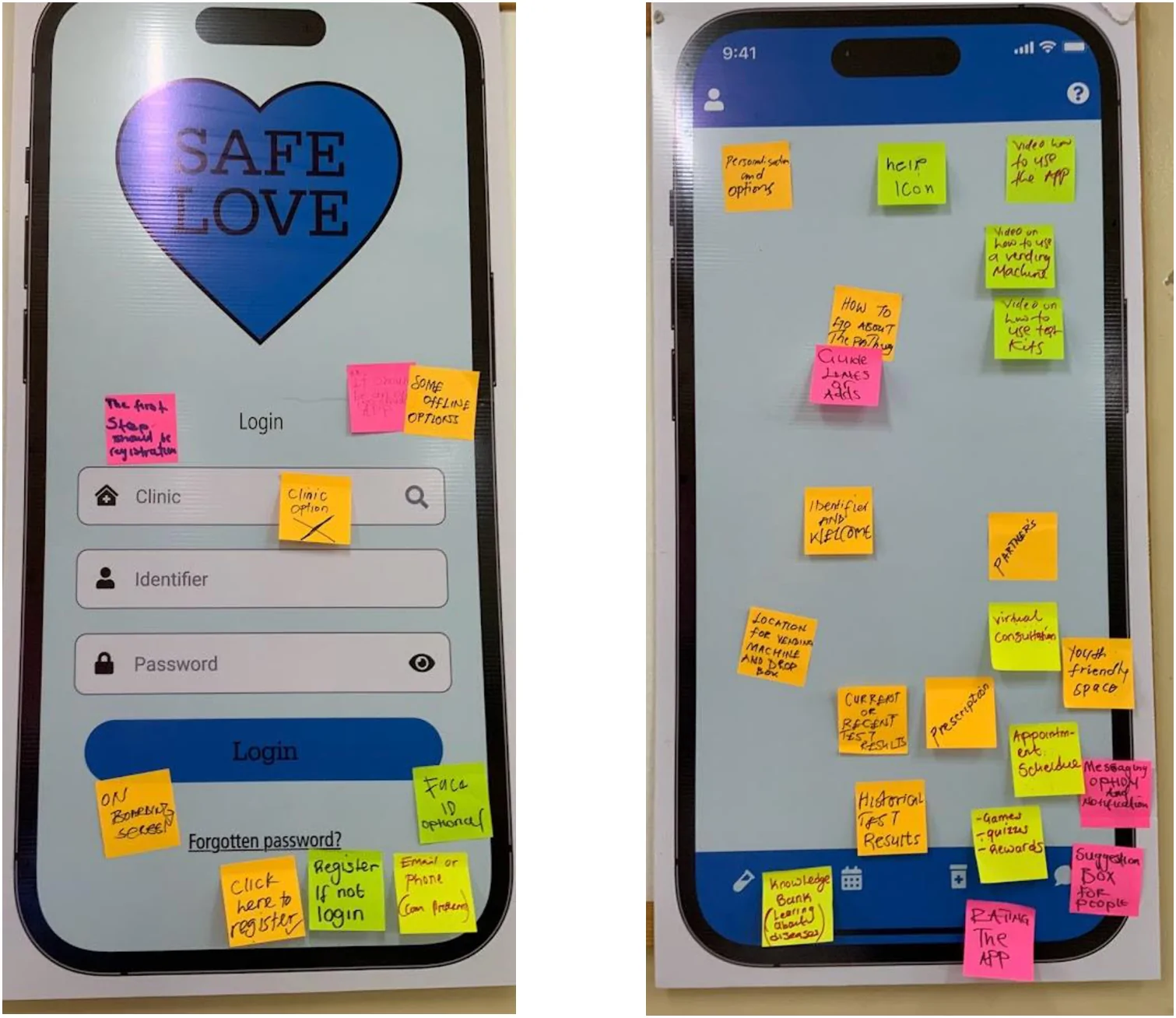
Enlarged images of app screens with ideas for app features and functionalities from Phase One co-design workshop.

To support youth-led ideation, participant co-designers were divided into two small groups for this part of the workshop. The role play method was extended with young people given the stimulus that they were now working for a company as part of an app design team and had to produce ‘the best’ features and functionalities for the eHealth Yabwela app. Each team was given two, large, ‘empty’ mobile phone screens (one ‘log in’ screen and one blank screen) and asked to write their ideas down on sticky notes and then place them on the enlarged screen (Fig 4). The log-in screen was explicitly included to prompt ideation on questions of data privacy and security. Teams were told that they should not feel limited by cost, timescales or what was technically possible at this stage, but instead to focus on “what does a young person living in Lusaka using the eHealth Yabwela pathway need and want from this app to make the pathway work?” (Workshop Facilitator prompt). At the end of this session, each team presented their design ideas to the other, narrating their choice of features and functionalities and explaining their reasoning for including them.

Team discussions and presentations were recorded and photos taken of the sticky notes each team included on the enlarged app screen images. The text from each sticky note was added to a spreadsheet of user requuirements. Once Phase One workshops were complete, the social science co-design lead collated and analysed app design requests. The pragmatist, analytical approach used aimed to generate development priorities whilst maximising transparency to youth co-designers and ensuring timely, actionable input for the technology development team. Over the course of four workshops, seven different teams (of between two and six co-designers) proposed a total of 223 ideas. Every idea was the product of intense, group discussion between youth co-designers, therefore every idea was documented and valued. The ‘Log-In Board’ generated an average of nine ideas per group and the ‘Blank Board’ an average of 23. The seven app design groups worked separately, therefore requests for the same functionalities or features were taken as indicators of consensus and therefore priority. For example, in Phase One all groups stated that the app should be available in different Zambian languages as well as English (Zambia’s national language). However, although prioritisation was consensus-driven, novel requests were not de-prioritised but treated as potentially important features which should be re-visited and discussed further. For example, having the facility to share app download details easily with friends and peers via a QR code embedded in the user’s profile was only listed once but was a feature that received a positive response from all Phase Two participants.

The technical team had three months to engage with and act on co-design outputs. Youth co-design priorities determined their work programme but time and budget constraints were also fundamental. A specification of which functionalities could be produced and made available for testing in Phase Two workshops was agreed with youth Co-design Leads along with the technical team’s explanations of why some functionalities were harder to build, or for data security reasons less advisable to pursue, than others. For example, embedding a QR code in a user profile was technically ‘quick’ and straightforward to achieve. However, creating a web-based, as well as mobile-phone based version, of the app would involve fundamental changes to the technical architecture and was therefore a much more complex and time-consuming proposal. With regard to data security, storing personal information locally on the mobile phone to allow young people to access their health information when offline was considered to be a risk, leading the academic research team to propose this should not be developed. This contrasted with young people’s accounts of the daily realities of mobile phone use, which involved finding places they could access Wi-Fi and reduce the need to pay for data. This tension prompted important discussions of sexual health data, security and privacy.

To ensure a thorough discussion of academic and technical team feedback, a ‘traffic light system’ was applied to the collated 223 requests where red items were those classed as unachievable in the project timeframe (or inadvisable for data security reasons), amber was achievable but required further discussion and green was achievable. Table 1 is an excerpt from the spreadsheet used to collate co-design and technical team feedback.

**Table 1:** Excerpt from collated and analysed user requested features and functionalities with agreed status and feedback from technology development team.

Online/ offline options	Status and technical team feedback
Ability to access app offline	Amber [more discussion needed]
**Account/ profile information/ personalisation**	
Personal information held in app stored on phone	Red [data security risk]
Choice of language very important (English official language; 72 others; high consensus, strong sentiment)	Green
Language and accessibility options throughout app and pathway	Green
i.e., by selecting preferences on app this carries through to vending machine interface and communications	Red [API development not achievable in project timeframe]
**Vending machine location and stock functionalities**	
Map of vending machine locations	Green
Which packs and products offered and current stock levels	Amber [could develop dummy database?]
**Deposit Boxes for completed samples**	
Map of deposit boxes	Green
Sample collection and delivery to lab recorded and reported	Amber [could develop dummy database?]
Sample ‘tracking data’ for app end user	Red [new functionality – not possible in timeframe]
**Referrals and appointments**	
Local health facility locations linked to Google map directions	Green
Appointment times and location Note: Very few clinical appointments with pre-stated time and location) in this care context.	Green
**Communications with healthcare workers and providers**	
SMS, WhatsApp, email, telephone preferences requested	Green

Where it was not possible to produce requested functionalities within the timeframe of the project these were completed ‘on paper’ as technical specifications that remained with Zambian partners for future development and implementation.

For example, following their visit to the container laboratory and discussions with the IT worker there, young people recognised a need for ‘audit and tracking’ functionalities that could record i.) if samples provided were sufficient and ii.) log what time samples had been deposited and subsequently arrived at the lab to determine if they were viable. Young people’s interest in these functionalities had not been anticipated and in previous projects, postal systems and laboratories interacted differently [64,65] so potential for this functionality had not been included in the reference technical architecture. Where UK research reports positive experience of using postal services to access home sampling and testing, young people and stakeholders in Lusaka were very aware that a breakdown in supply, transport and laboratory logistics would render the pathway ineffective and so the value of using digital technology for sample tracking and monitoring was clear. Whilst there was not time to technically develop this functionality in-project, it was specified ‘on paper’ and rated ‘Amber’ in the user requirements log, ready for future development.

In another example, discussion concerning what products the vending machine should contain led to recognition that altering mechanical design would be more costly and time consuming than altering the digital interface design, so for the purposes of pilot work, this component was treated as ‘fixed’. It was also recognised that the products contained within boxes needed to make sense clinically and in terms of available laboratory capabilities, but beyond this, products could be flexibly organised and grouped together. Therefore, when women co-designers expressed concern about the idea of vaginal self-sampling for STIs, clinical co-researchers were able to inform them that a urine sample was also viable, which made this option more acceptable to them. In this way, researchers learned about young women’s preferences, identified education potential and embedded acceptability into the service specification, instead of encountering this question as a barrier to women’s use post-implementation.

In addition to the smartphone application, a prototype specification of the clinic or laboratory worker-facing web application was shared with participants and a laboratory IT worker. Based on their feedback a prototype design for this component was confirmed. An aim of the web application was that after results have been processed at the laboratory and before they are sent to a young person’s phone they can be reviewed and a choice made about whether to send the results, a message or both.

### 4.3 User testing the pathway and stakeholder engagement

In Phase Two, a further four workshops were organised. Following the same co-production principles, Co-design Leads (n = 7) met first and co-created the format for the subsequent three workshops (24 attendees). The aim of Phase Two workshops was to reproduce the eHealth Yabwela pathway and user journey co-designed in Phase One but this time using functional prototype technologies. Ultimately, it was possible to create four digital components of the pathway: the clinical or lab worker-facing web application; the API linking this to the smartphone application; a test or ‘dummy’ database of results and user profiles; an API linking the dummy database to the smartphone application; and the smartphone application. It was not possible to co-design the vending machine digital interface in the timeframe of the project.

Usable on either Android or Apple operating systems, the eHealth Yabwela test app was made available for download from Google Play and the App Store and after re-creating the six installations around the room and rehearsing the steps on the pathway, youth co-designers downloaded the app using a QR code or by entering a short web address displayed on the vending machine banner. On doing so, the onboarding screens and app registration process were noted by Phase Two testers and co-designers as requiring further work. However, the option to switch between languages so that the app user interface appeared in Bemba or Nyanja, as well as English, was tried out at this point and highly valued by some testers. Others were not so sure noting that some words (such as ‘loading’ the word used in English when there is a technical delay in a screen appearing) were hard to translate and longer word lengths led to the interface layout looking less clear.

Once logged into the smartphone app, participants were able to enter a code from a sample deposit envelope, thereby uniquely linking their test profile to the sample. Participants were then invited to play the role of ‘Laboratory IT worker’ whereby they logged into the prototype web application and viewed where the test profiles and samples just entered were now visible along with associated test results. Via this clinical or laboratory worker-facing application they could review and where appropriate send results to app users along with a standardised or edited text or WhatsApp message (depending on app user communication preferences). Finally, participants turned back to their phones to see the dummy test results for the sample they had logged appear in the smartphone application.

In parallel to Phase Two workshops, three focus groups were held with community, policy and healthcare worker stakeholders. These three hour workshops introduced stakeholders to the pathway and asked for their opinions, feedback and advice on its viability, feasibility and acceptability. Beyond this, stakeholder workshops were relatively unstructured so discussion points stemmed from participant interest. Common questions concerning where to locate vending machines, how to support young people who do not have smartphones and how to embed treatment pathways into existing youth-friendly services in local health facilities emerged as focal conversation points. FGD participants perceived smartphone use among young people to be widespread and recognised the potential skill and enthusiasm young people had to offer digital health innovation design processes. They also reflected positively on the attentiveness young people had directed towards questions of digital inequity and the importance they had placed on designing equitable access to healthcare. Community and policy stakeholders exhibited more concerns about ensuring security and avoiding exploitation of community-based vending machines than workshop participants did. They also directed focus toward the cost of vending machine products and the associated operational costs of maintaining and servicing machines.

## 5. Discussion

In contrast to research methods aimed at producing generalisable findings, the research strategy adopted here purposefully *located* the innovation process, focusing researcher and developer attention on young people’s experience of phone use and accessing care in Lusaka, Zambia. Via this method, research and innovation were conceptualised as taking place within a socio-technical flow over time [30,31]. According to this theoretical framing, generalisable knowledge and re-usable technologies are *made* viable and capable of impacting health access inequity by combining them with located knowledge and lived experience. Co-production creates the ethical conditions needed for place-focused engagement and deliberation to occur. From this foundation, co-design methods supported *in situ,* material engagement in prototype design and testing. These made complex, less tangible digital and data elements and less known elements (like vending machines) available for inquiry and deliberation. Alongside these methods young people spoke about what it is like to access SRH support in Lusaka and about the different ways they use mobile phones in their lives. This ideation flowed into their smartphone app designs which focused on i.) making the intervention usable through inclusion of maps, information on youth-friendly local services, contacts for local SRH peer advisors and sample tracking functionalities and ii.) making the intervention accessible through functionalities to support communication needs and local language options. It also led to problematisation of health access and digital inequity which produced complementary designs that were deliberately less dependent on data, energy infrastructure and smartphone ownership. Through their peer-based reflection on health, smartphone journeys and vending machines they produced a ‘pathway’ that could signal “there *is* help” (Co-design participant) and “show the way” (Co-design participant) in moments when participants might not know "which way they can turn for the best" (Co-design participant). They drew on this empathy-led, peer-based co-ideationto specify requirements for digital technologies and functionally tested the end results which youth co-designers valued and were proud of (“*We* did this” Co-design Lead) and for which they chose to advocate by producing a short film.

We argue that place-based co-production has ethical significance and practical utility in research and innovation for three reasons. Firstly, co-production provides a means of engaging with located conditions of healthcare access constructively and ethically such that meaningful change can be specified and accountably pursued. Secondly, for implementation planning to be embedded into pilot projects, infrastructural conditions and institutional contexts of implementation sites must be known and requirements anticipated. These conditions are localised and even where partially specified, require detailed dialogue with people who live and work in places to be reliably known. Co-production guards against more instrumental, extractive forms of research and technology piloting that have no clear agenda for engaging in this dialogue and ensuring the value of research and innovation can be mobilised in the places it is conducted. Thirdly, the current pace of digital and medical innovation is such that all stakeholders (professional, policy makers and public) in processes of health-oriented, socio-technological change require time and space to understand, problematise and reach consensus on how these changes will manifest in their setting.

Youth-led co-production supported co-design priorities to flow from place-based lived experience into digital service design. It achieved this via an emergent process of pragmatic inquiry and experiential learning using arts-based methods. Young people took different roles and responsibilities in this process. They were leaders (“the bosses”) as well as drivers, laboratory workers, app designers and ultimately evaluators of the proposed intervention, signalling at the end of each workshop whether or not they considered the proposed pathway acceptable, usable and feasible (100% did – but with caveats). The conditions that would ordinarily problematise and exclude an under-served group from having decision-making power and the privilege required to occupy the foreground of problem identification, rationalisation and priority-setting, were re-framed through this process. By contrast, the HCD cycle was pre-planned and highly structured providing a common, interdisciplinary framework for the wider project research team to organise their activity around, crucially: i.) pausing prior to technology development to allow time for co-production to begin ii.) producing technical specifications that demonstrated fidelity to co-design activity iii.) waiting for user testing of functional prototypes to occur before iv.) implementing user testing feedback and creating paper-based specifications where technology was not feasible.

When combined with co-design methods, we show how co-production forms an effective basis for developing collective understanding of complex, integrated healthcare interventions. Frameworks for flexibly combining and recombining technologies using modular approaches to reusable technical architectures have long been recognised as fundamental to sustainable, scalable digital health service design [27,66]. Similarly, integrated health service design research has evidenced how multi-component interventions, incorporating a range of sites (home, community-based, health facility) have potential to impact health outcomes for people [67]. We show that place-based, co-design methods supplement and create opportunity for deliberation on *in situ* implications of flexibly combining sites, spaces, locations, devices, digital technologies, machines and infrastructural capabilities. In our research, such methods reduced barriers to engagement with complex innovation and elicited whole system, health equity thinking. To ensure dialogue was accountably translated into action, the flow of technology development was phased and transparently conducted such that young people *saw* developers *wait* for their requirements, were able to *discuss* with them what requirements should progress and *test* the functional prototypes this produced. Clinical and budgetary rationales were transparently shared with young people so that they could assess decisions in dialogue with developers and clinicians. Whilst this is one example, the process of requirements gathering, specification, development and testing is a globally recognised standard in software engineering and systems development [68]. Therefore, proposing methods that impact this standard holds implications for people-centred digital design at any scale. From large-scale trials and policy-led processes to small-scale public involvement, such methods can direct collective focus toward accountability, anchorage in local systems and attunement to local needs and contexts.

In the case of HIV and STIs, capacity for aetiological diagnosis holds significant implications for people’s lived experience of physical symptoms, infertility, onward transmission and the profound anxiety and stigma SRH health concerns can result in. As we have argued, addressing these issues in sustainable, cost-effective ways requires frameworks for mobilising knowledge that recognise the significance of generalisable knowledge and reusable technologies, yet engage substantively with people and places. For example, in the case of Zambia, the impacts of electricity outages resulting from load shedding affect all aspects of life [69,70]. Although conditions have improved, outages of 20 hours per day were recorded for domestic consumers between March and December 2024 [71]. Whilst workarounds can ensure personal mobile devices remain charged, prolonged outages affect mobile network towers, resulting in intermittent connectivity and reduced network performance, particularly in areas of high population density. Creating mobile phone dependent services in a broader global context of climate change and energy insecurity requires that intervention designs account for the effects of climate on energy supply and difference in place-based experiences of infrastructure.

Next steps for this research include a commitment to implementation of the eHealth Yabwela pathway in Zambia and associated evaluation research that can evidence its impact on SRH outcomes, in particular, HIV testing, diagnosis and reduction in rates of curable STIs. The potential of integrated frameworks for introducing opportunities for testing, diagnosis, linkage to care and treatment into community-health facility care pathways are well-established [72] as are their capacity for adaptation in response to outbreaks and pandemic [73,74]. Containerised laboratory infrastructures with integrated digital and diagnostic capabilities, alongside point-of-care, self-testing and self-sampling technologies, are undergoing rapid development as part of broader trends toward decentralised and near-patient healthcare delivery [75]. These have potential to encompass a range of communicable and non-communicable diseases [76,77] enabling co-design of more holistic service models. In addition, public involvement and debate will be required on responsible integration of artificial intelligence (AI) into integrated health intervention designs including the potential of federated learning [78]. Whilst prototyping cannot account for the complexities of implementation, it can provide valuable time and space for involvement, deliberation and place-based attunement as we have shown. Digital innovation is notoriously subject to a productivity paradox [79] where cost benefits are hard to specify as recent developments with Artificial Intelligence have shown [80]. Compared to the cost of digital innovation, costs of co-production and co-design are very low. However, as a method whose immediate aim is to re-constitute involvement in decision-making and re-work processes of collaboration such that longer term social change can be actualised, co-production is less amenable to conventional, short-term, outcome-based measurement. Such approaches infer timeframes and causal effects that are frequently incommensurate with complex social change. Alternative approaches to defining and evidencing what constitutes an accountable approach to research and innovation are required if valuable stakeholder time and commitment is not to be misdirected via attempts to meet the requirements of poorly conceived evaluation regimes [81]. Social design theorists argue that processes that “make publics” who are able to deliberate, problematise and make decisions together are themselves a common social good essential to responsible design, research and innovation.

WHO recommendations [82] on digital innovation for health systems strengthening and universal health coverage are critical of digital innovation that does not move beyond prototype phases or that leads to a “proliferation of short-lived implementations” that have potential to “inappropriately divert resources from alternative, non-digital approaches”. A people-centred, whole systems approach that requires consideration of low-tech and no-tech initiatives and purposefully includes community-based and outreach modalities is crucial to achieving equity of access. Through its participatory focus on generating value exchange, rather than extractive, short term consultation, we argue co-production has potential to positively impact innovation processes and the people involved in them. Young people universally described their participation in eHealth Yabwela as a valuable learning experience that supported them to gain understanding, not only about sexual health and self-testing/sampling, but also: health service design; software design; technology development processes; data privacy; and medical laboratory testing infrastructure. Whilst digital health innovation processes are rightly focused on realising medical and technological capabilities, place-based co-production can contribute to them by providing a means of orienting activity toward people and places. For youth co-designers in our project, their motivation for creating the eHealth Yabwela pathway came from a recognition that young people can feel worried and alone when they encounter SRH concerns. Instead of not knowing which way to turn at these times, they wanted them to find a path to trusted advice, peer support and youth-friendly healthcare workers. In addition, they wanted to bring recognition to the role that smartphones now form play in many young Zambian people’s lives. They did this whilst placing digital and health equity concerns for their peers at the forefront of discussions and instinctively directing inclusive pathway design. They valued the process of being involved in digital health innovation co-production. As one Co-design Leader said “This way you learn. The other way is sitting, listening, without saying *your* ideas. This way they *hear* you.” (Co-design Lead Workshop).
