# Supplementary material for: Co-producing access to care with young people living in Zambia through digital and vending machine-enabled innovation in sexual and reproductive health: A place-based, human-centred design research approach
Source: PLoS One. 2026 Aug 27;21(8):e0356398. doi: 10.1371/journal.pone.0356398 (PMC13521473; doi:10.1371/journal.pone.0356398)
Supplement: Supplementary file 1 [file pone.0356398.s002.docx]

20^th^ May 2026

Dear Dr Fuller

My co-authors and I would like to thank you and our anonymous reviewer sincerely for your recommendations. We have done our utmost to address each point and we include with this letter a ‘Revised manuscript with track changes’ and a ‘clean’ copy of our manuscript for your consideration.

Outside the revisions specified, we would like to request the addition of a co-author. Our laboratory colleague Deborah Milimo-Kabula played a vital role in demonstrating container laboratory capabilities but was not originally part of the team who wrote this article. She has now read and contributed to the article and we would like to rectify our original mistake by including her as co-author now.

1.Please ensure that your manuscript meets PLOS ONE's style requirements, including those for file naming

The full manuscript has been reviewed to ensure that it aligns to PLOS ONE’s style requirements.

2. Regarding our financial disclosure statement, we can confirm that the funders had no role in the research. As directed, we include the statement suggested here (in our cover letter) and would be grateful if you would add the amended statement to our submission:

"“Medical Research Council Public Health Intervention Development Fund (MR/X503058/1) £136,669. The funders had no role in study design, data collection and analysis, decision to publish, or preparation of the manuscript."

3. Regarding the competing interests statement, we have checked and can confirm that we are compliant with all PLOS ONE policies on sharing data and materials. The statement has been amended as directed and is included in full below. We would be grateful if you would add the amended statement to our online submission.

"I have read the journal's policy and the authors of this manuscript have the following competing interests: Dr Jenny Whetham is a non-salaried Director of the not-for-profit EmERGE mHealth Ltd. who were the digital innovation partner for this project. EmERGE mHealth Ltd was set up to ensure sustainability of a previous research project output funded by the European Union’s Horizon 2020 research and innovation programme grant number: 643736. This does not alter our adherence to PLOS ONE policies on sharing data and materials.”

4. You noted that “funding information should not appear in any section or other areas of your manuscript.[…]” and requested “Please remove any funding-related text from the manuscript.”

Information about our Medical Research Council funding was included on Line 108, p.5 and this has been removed.

“Funding-related text” was taken to mean any sentence that referenced our funding. Therefore, amendments were also made to the following lines in the manuscript:

Line 109 | p.5

Line 359 | p.14

Line 361 | p.14

Line 652 | p.26

Line 689 | p.27

5. Regarding data sharing we produced ‘a minimal anonymised dataset’ within our team for discussion on whether there was a method for sharing data that allowed us to meet ethical requirements. On reflection and with regret we agreed that sharing a minimal dataset is not possible. Our ethical objective in this research was to produce specifications and requirements that our Zambian partner could mobilise in future research for the benefit of their healthcare system. To preserve the value of the requirements we developed for them we have taken not to publish our underlying data. We therefore direct you to page 17 of our research protocol submitted to UNZABREC and approved 4^th^ Jan 2023:

## “7.3 Data sharing

Data will not be shared with anyone outside of the project team and organizations hosting the research.”

Data requests can be sent to:

**UNIVERSITY OF ZAMBIA BIOMEDICAL RESEARCH ETHICS COMMITTEE**

Ridgeway Campus

P.O. Box 50110

Lusaka, Zambia

E-mail: unzarec@unza.zm

6. “Your ethics statement should only appear in the Methods section of your manuscript.”

Our ethics statement appears in the Methods section and nowhere else in the manuscript.

7. Regarding content permission for images used in Figures 2 and 3, we have substituted 3 images in these figures. All images are now owned by our partner Zambart apart from the image of the mobile phone screen (owned by EmERGE mHealth) and the post box published (free content published by Pixabay). We have provided a list of all images with sources and two content permission forms (using PLOS One template) which Zambart and EmERGE mHealth have signed respectively.

8. We notice that your supplementary figures are uploaded with the file type 'Figure'. Please amend the file type to 'Supporting Information'. Please ensure that each Supporting Information file has a legend listed in the manuscript after the references list.

All four Figures have now been uploaded with the File Type ‘Supporting Information’ and a legend listing each one included after the reference list.

9. The anonymous reviewer did not recommend that we cite any specific published works

Responses to Reviewer 1 Comments

We thank the reviewer sincerely for their comments on our paper. We are grateful for their observations and their recognition that our paper “will be useful to others thinking about digital health and integrated care delivery systems.”

The reviewer includes two comments regarding the analysis process stating that the analytical method should be made “explicit and detailed in the methods section”.

These points have been addressed in the penultimate paragraph of the methods section (Section 3).

C. The reviewer supports our decision to not share focus group data and has noted that we have shared our visual materials. They would like us to share textual data. We have explained our reason for not doing so in our reply to the PLOS One Editor on page 1.

D. The paper has been edited for conciseness, clarity, and flow. Language that was used within the co-design process which the reviewer considers colloquial (“stations,” “blank boards,” and “technical architecture”) has been removed, clarified or defined.

## Introduction

The reviewer made 2 recommendations regarding the introduction which have been addressed as follows:

1. The flow of the argument has been improved and the detailed instructions provided by the reviewer followed. This has led to material from the first and second paragraph being combined.

2. The last paragraph of the introduction has been expanded to include further articulation of the wider implications of the research.

## Theoretical Framework:

1. **The anonymous reviewer asked that Line 134 should be “dropped or made meaningful”. The sentence has been retained and added to as follows because the reference it makes to the 2022 systematic review on knowledge mobilisation is significant to the main argument.**

In the context of healthcare, this is particularly relevant where intervention design calls for different types of knowledge to be mobilised, yet not all knowledge types have equal status^36^.

2**. Line 139 & 140 - Do you mean co-production as used in public health? If so, please clarify. Otherwise this statement is accurate but denies the comprehensive world of co-production. The rest of this paragraph would benefit from simplification through removal of jargon and borrowed text.**

We have added some key references into our definition of co-production which should show the range and breadth of its use, prior to its adoption in healthcare. We have removed words that could be considered jargon. The phrase ‘borrowed text’ is unfamiliar to us. We understand from this that the text is considered unclear, but as a qualitative researcher, use of direct quotations is an important practice that grounds definitions and concepts in precise terms. We have therefore reduced but not removed entirely, quoted text from this paragraph.

**3. The anonymous reviewer notes: “Paragraph starting 148 - Earlier you already talked of 'in situ making' which lends itself to place-based. Place-based is not a new term nor does it lend itself to an imagination of the 'complementary role that co-production can play in translating science and engineering knowledge.' Consider scaling this statement down accordingly.”**

The sentence referred to has been removed.

**4. The anonymous reviewer notes: “Paragraph starting 148 - This whole paragraph could also benefit from simplification through removal of jargon and borrowed text. Consider integrating into the paragraph that follows to make your case stronger”**

Arguments made in the text referred to have been simplified and the two paragraphs combined.

**Research Design**

1. Line 187 - the UNZABREC approval number has been provided

**2. The anonymous reviewer asks: How were focus group discussions documented and analysed?**

They were recorded and transcribed. A full description of the analytical process is provided in the penultimate paragraph of section 3. Please note, as this was multi-methods, qualitative research that involved the production on technically functional prototypes, in-depth, qualitative analysis of transcripts was not conducted due to time and resource constraings

**3. The anonymous reviewer asks: How were the data from the workshops documented and analysed?**

Workshops were recorded and transcribed or else detailed notes taken. Further information on the workshop findings have been included at the end of section 4.3

**Findings**

**The anonymous reviewer notes: “1. The paragraph on FGDs is so cursory that it begs its utility in this paper; yet it is important to your stated aim to assess stakeholder acceptability in this manuscript. Perhaps remove all reference to this throughout or ideally, make explicit these findings as its of import and interest on whether young voices find resonance with other stakeholders.”**

Further information on the workshop findings have been included at the end of section 4.3. As noted previously, as multi-methods, innovation research in-depth qualitative comparison of workshops and FGDs was not possible within the time and resources available.

**Discussion**

**The anonymous reviewer makes 3 points. “1. The discussion is unnecessarily long and includes findings. Consider tying back more clearly to what you intend this paper to do in about 4-5 paras on use of participatory design and place-based engagement, and the lived reality of syndromic management, little lab capacity/cost, the cost of maintaining/servicing technology, load shedding, cost of managing etc.**

**2. Suggest a clear paragraph on next steps: What needs to happen next? Implementation trials? Integration with HMIS? Assessment of cost-effectiveness? This would show the pathway from prototype to health system uptake.**

**3. Tie back to the wider implication of sharing your approach - what processes are transferrable, to whom, for what purpose? How does your experience resonate with what others report? What is new?”**

Thank you for these comments which were very focusing for us. The Discussion is now 2534 words and is now and is now 1350 words (approx. 1200 words shorter). Any findings have been removed apart from those offering a brief summation of research outcomes in the first paragraph. There are now 6 paragraphs in total. They follow the pattern: summary of findings and approach; impact of the conceptual approach used; contribution; wider implications; next step; concluding paragraph

4. Line 529 – The citation # for Hayes et al has been provided

Minor Comments

All Minor Comments identified by the reviewer have been addressed as follows;
1. All commas in citations are now in superscript
2. The format of all alpha numeric labels has been changed to #) not #.) throughout

3. Line 134 – comma deleted

4. Line 137 - period inserted after superscript 38
5. Line 199 – Comma inserted between the numbers 48 and 49 in design (HCD) cycle48 49
6. Line 213 - 'In addition' deleted.
7. Line 314 – comma added after In Zambia
8. Line 331 - 'and onto health facilities' re-phrased to ‘and then to local health facilities’

9. Line 356 - 'on' removed

10. Line 363 - Comma included after (2015-2020)
11. Line 386-87 closing double-speech mark added after ?

12. Line 401 (now Line 402) – comma added before therefore.
13. Throughout numbers 1-9 have been spelled out.
14. Line 429 – Sentence edited to improve grammar
15. Line 446-447 - Repeated item ‘API’ checked and clarified

Thank you once again for these insightful comments and corrections.

Yours sincerely,

Dr Mary Darking (Corresponding Author)
