# Supplementary material for: Co-producing access to care with young people living in Zambia through digital and vending machine-enabled innovation in sexual and reproductive health: A place-based, human-centred design research approach
Source: PLoS One. 2026 Aug 27;21(8):e0356398. doi: 10.1371/journal.pone.0356398 (PMC13521473; doi:10.1371/journal.pone.0356398)
Supplement: Peer review history

# Peer review: Decision Letter 0

**DOI:** 10.1371/journal.pone.0356398.r001

**Authors:** Sebastian Suarez Fuller

26 Mar 2026

Dear Dr. Darking,

Please submit your revised manuscript by May 10 2026 11:59PM. If you will need more time than this to complete your revisions, please reply to this message or contact the journal office at plosone@plos.org. When you're ready to submit your revision, log on to https://www.editorialmanager.com/pone/ and select the 'Submissions Needing Revision' folder to locate your manuscript file.

- A letter that responds to each point raised by the academic editor and reviewer(s). You should upload this letter as a separate file labeled 'Response to Reviewers'.
- A marked-up copy of your manuscript that highlights changes made to the original version. You should upload this as a separate file labeled 'Revised Manuscript with Track Changes'.
- An unmarked version of your revised paper without tracked changes. You should upload this as a separate file labeled 'Manuscript'.

If you would like to make changes to your financial disclosure, please include your updated statement in your cover letter. Guidelines for resubmitting your figure files are available below the reviewer comments at the end of this letter.

If applicable, we recommend that you deposit your laboratory protocols in protocols.io to enhance the reproducibility of your results. Protocols.io assigns your protocol its own identifier (DOI) so that it can be cited independently in the future. For instructions see: https://journals.plos.org/plosone/s/submission-guidelines#loc-laboratory-protocols. Additionally, PLOS ONE offers an option for publishing peer-reviewed Lab Protocol articles, which describe protocols hosted on protocols.io. Read more information on sharing protocols at https://plos.org/protocols?utm_medium=editorial-email&utm_source=authorletters&utm_campaign=protocols.

As the corresponding author, your ORCID iD is verified in the submission system and will appear in the published article. PLOS supports the use of ORCID, and we encourage all coauthors to register for an ORCID iD and use it as well. Please encourage your coauthors to verify their ORCID iD within the submission system before final acceptance, as unverified ORCID iDs will not appear in the published article. *Only*  the individual author can complete the verification step; PLOS staff *cannot*  verify ORCID iDs on behalf of authors.

We look forward to receiving your revised manuscript.

Kind regards,

Sebastian Suarez Fuller, PhD

Academic Editor

PLOS One

Journal Requirements:

When submitting your revision, we need you to address these additional requirements.

1.Please ensure that your manuscript meets PLOS ONE's style requirements, including those for file naming. The PLOS ONE style templates can be found at

https://journals.plos.org/plosone/s/file?id=wjVg/PLOSOne_formatting_sample_main_body.pdf and

https://journals.plos.org/plosone/s/file?id=ba62/PLOSOne_formatting_sample_title_authors_affiliations.pdf

2. Thank you for stating the following financial disclosure:

“Medical Research Council Public Health Intervention Development Fund (MR/X503058/1) £136,669”

Please state what role the funders took in the study.  If the funders had no role, please state: "The funders had no role in study design, data collection and analysis, decision to publish, or preparation of the manuscript."

If this statement is not correct you must amend it as needed.

Please include this amended Role of Funder statement in your cover letter; we will change the online submission form on your behalf.

3. Thank you for stating the following in the Competing Interests section:

“I have read the journal's policy and the authors of this manuscript have the following competing interests: Dr Jenny Whetham is a non-salaried Director of the not-for-profit EmERGE mHealth Ltd. who were the digital innovation partner for this project.

EmERGE mHealth Ltd was set up to ensure sustainability of a previous research project output funded by the European Union’s Horizon 2020 research and innovation programme grant number: 643736”

Please confirm that this does not alter your adherence to all PLOS ONE policies on sharing data and materials, by including the following statement: "This does not alter our adherence to  PLOS ONE policies on sharing data and materials.” (as detailed online in our guide for authors http://journals.plos.org/plosone/s/competing-interests).  If there are restrictions on sharing of data and/or materials, please state these. Please note that we cannot proceed with consideration of your article until this information has been declared.

Please include your updated Competing Interests statement in your cover letter; we will change the online submission form on your behalf.

4. Please note that funding information should not appear in any section or other areas of your manuscript. We will only publish funding information present in the Funding Statement section of the online submission form. Please remove any funding-related text from the manuscript.

5. We note that you have indicated that there are restrictions to data sharing for this study. PLOS only allows data to be available upon request if there are legal or ethical restrictions on sharing data publicly. For more information on unacceptable data access restrictions, please see http://journals.plos.org/plosone/s/data-availability#loc-unacceptable-data-access-restrictions.

Before we proceed with your manuscript, please address the following prompts:

a) If there are ethical or legal restrictions on sharing a de-identified data set, please explain them in detail (e.g., data contain potentially identifying or sensitive patient information, data are owned by a third-party organization, etc.) and who has imposed them (e.g., a Research Ethics Committee or Institutional Review Board, etc.). Please also provide contact information for a data access committee, ethics committee, or other institutional body to which data requests may be sent.

b) If there are no restrictions, please upload the minimal anonymized data set necessary to replicate your study findings to a stable, public repository and provide us with the relevant URLs, DOIs, or accession numbers. For a list of recommended repositories, please see

https://journals.plos.org/plosone/s/recommended-repositories. You also have the option of uploading the data as Supporting Information files, but we would recommend depositing data directly to a data repository if possible.

We will update your Data Availability statement on your behalf to reflect the information you provide.

6. Your ethics statement should only appear in the Methods section of your manuscript. If your ethics statement is written in any section besides the Methods, please move it to the Methods section and delete it from any other section. Please ensure that your ethics statement is included in your manuscript, as the ethics statement entered into the online submission form will not be published alongside your manuscript.

7. We note that Figure 2 and 3 in your submission contain copyrighted images. All PLOS content is published under the Creative Commons Attribution License (CC BY 4.0), which means that the manuscript, images, and Supporting Information files will be freely available online, and any third party is permitted to access, download, copy, distribute, and use these materials in any way, even commercially, with proper attribution. For more information, see our copyright guidelines: http://journals.plos.org/plosone/s/licenses-and-copyright.

We require you to either (1) present written permission from the copyright holder to publish these figures specifically under the CC BY 4.0 license, or (2) remove the figures from your submission:

1. You may seek permission from the original copyright holder of Figure 2 and 3 to publish the content specifically under the CC BY 4.0 license.

We recommend that you contact the original copyright holder with the Content Permission Form (http://journals.plos.org/plosone/s/file?id=7c09/content-permission-form.pdf) and the following text:

“I request permission for the open-access journal PLOS ONE to publish XXX under the Creative Commons Attribution License (CCAL) CC BY 4.0 (http://creativecommons.org/licenses/by/4.0/). Please be aware that this license allows unrestricted use and distribution, even commercially, by third parties. Please reply and provide explicit written permission to publish XXX under a CC BY license and complete the attached form.”

Please upload the completed Content Permission Form or other proof of granted permissions as an "Other" file with your submission.

In the figure caption of the copyrighted figure, please include the following text: “Reprinted from [ref] under a CC BY license, with permission from [name of publisher], original copyright [original copyright year].”

2. If you are unable to obtain permission from the original copyright holder to publish these figures under the CC BY 4.0 license or if the copyright holder’s requirements are incompatible with the CC BY 4.0 license, please either i) remove the figure or ii) supply a replacement figure that complies with the CC BY 4.0 license. Please check copyright information on all replacement figures and update the figure caption with source information. If applicable, please specify in the figure caption text when a figure is similar but not identical to the original image and is therefore for illustrative purposes only.

8. We notice that your supplementary figures are uploaded with the file type 'Figure'. Please amend the file type to 'Supporting Information'. Please ensure that each Supporting Information file has a legend listed in the manuscript after the references list.

9. If the reviewer comments include a recommendation to cite specific previously published works, please review and evaluate these publications to determine whether they are relevant and should be cited. There is no requirement to cite these works unless the editor has indicated otherwise.

Additional Editor Comments:

Apologies with the delays in getting our decision back to you. If you choose to revise and resubmit, please attend to all the comments made by reviewer 1 in your revision.

[Note: HTML markup is below. Please do not edit.]

Reviewer's Responses to Questions

**Comments to the Author**

1. Is the manuscript technically sound, and do the data support the conclusions?

Reviewer #1: Yes

**********

2. Has the statistical analysis been performed appropriately and rigorously?

Reviewer #1: N/A

**********

3. Have the authors made all data underlying the findings in their manuscript fully available?

The PLOS Data policy

Reviewer #1: No

**********

4. Is the manuscript presented in an intelligible fashion and written in standard English?

Reviewer #1: No

**********

Reviewer #1: This is an important paper sharing practical steps and experiences with a very futuristic view for Zambia given current geopolitical, structural constraints. Overall, the paper captures all required material except findings from FGDs which is dealt with very cursorily. Once edited for conciseness, clarity, and flow, and the wider utility made more apparent, this paper will be useful to others thinking about digital health and integrated care delivery systems.

A. Is the manuscript technically sound, and do the data support the conclusions?

Yes, the findings convincingly demonstrate that the co-produced innovation process, grounded in the theory of Sociotechnical Transformation and supported by a participatory analytic approach, effectively embedded youth voices and place-specific priorities into both the conceptual and technical architecture of the eHealth Yabwela pathway. The process moved beyond consultation to active co-creation, resulting in functional digital prototypes that reflect the lived realities and preferences of young people in Lusaka.

B. Has the analysis been performed appropriately and rigorously?

I believe so; however, these need to be made explicit and detailed in the methods section.

C. Have the authors made all data underlying the findings in their manuscript fully available?

The data is not available though visual materials are included. The reasons for not sharing textual data are not sound as only names would need redacting. However, I do agree with qualitative/in-depth data being unavailable - qualitative data taken out of context without an emic view and inclusion of local researchers is subject to faulty interpretation and is extractive.

D. Is the manuscript presented in an intelligible fashion and written in standard English?

The paper is easy to follow but the English is colloquial rather than professional; this would be acceptable had it been written by the co-design leaders. More experienced and senior authors should edit for conciseness, clarity, and flow. Terms such as “stations,” “blank boards,” and “technical architecture” are used without clarification/definition.

Below are some suggested revisions that I believe will further strengthen the paper.

Introduction:

The flow of the argument can improved as below:

1. The first paragraph is misplaced and appears to be hastily put together. I suggest you replace the first sentence with that from your 2nd para and argue why testing is a solution. The last sentence refers to stigma and discrimination without cogent evidence of how it acts and what might be a solution.Combining your first and second para will address this as well as remove a very slim 2nd para.

2. Please expand on your last paragraph. What is the wider implication of sharing your approach? Why should your reader care about what you did for a small sub-population in one country with the intent of getting very localised findings?

Theoretical Framework:

1. Line 134 - the last sentence either needs to be dropped or made meaningful.

2. Line 139 & 140 - Do you mean co-production as used in public health? If so, please clarify. Otherwise this statement is accurate but denies the comprehensive world of co-production. The rest of this paragraph would benefit from simplification through removal of jargon and borrowed text.

3. Paragraph starting 148 - Earlier you already talked of 'in situ making' which lends itself to place-based. Place-based is not a new term nor does it lend itself to an imagination of the 'complementary role that co-production can play in translating science and engineering knowledge.' Consider scaling this statement down accordingly.

4. Paragraph starting 148 - This whole paragraph could also benefit from simplification through removal of jargon and borrowed text. Consider integrating into the paragraph that follows to make your case stronger.

Research Design

Thank you for a detailed, transparent section!

1. Line 187 - please provide UNZABREC approval number

2. How were focus group discussions documented and analysed?

3. How were the data from the workshops documented and analysed?

Findings -

1. The paragraph on FGDs is so cursory that it begs its utility in this paper; yet it is important to your stated aim to assess stakeholder acceptability in this manuscript. Perhaps remove all reference to this throughout or ideally, make explicit these findings as its of import and interest on whether young voices find resonance with other stakeholders.

Discussion -

1. The discussion is unnecessarily long and includes findings. Consider tying back more clearly to what you intend this paper to do in about 4-5 paras on use of participatory design and place-based engagement, and the lived reality of syndromic management, little lab capacity/cost, the cost of maintaining/servicing technology, load shedding, cost of managing etc

2. Suggest a clear paragraph on next steps: What needs to happen next? Implementation trials? Integration with HMIS? Assessment of cost-effectiveness? This would show the pathway from prototype to health system uptake.

3. Tie back to the wider implication of sharing your approach - what processes are transferrable, to whom, for what purpose? How does your experience resonate with what others report? What is new?

4. Line 529 - Please provide citation # for Hayes et al

Minor Comments:

1. Throughout the document, commas in citations should be in superscript, for e.g., Line 60: their social and economic wellbeing across the lifecourse 3,4. - The comma should be in superscript.

2. Line 110 - Consider using #) not #.) throughout; also see alpha.) as in a.) etc throughout

3. Line 134 - exclusions are,34,35. Remove , after are.

4. Line 137 - You need a period after superscript 38

5. Line 199 - Please put , between the numbers 48 and 49 in design (HCD) cycle48 49

6. Line 213 - Consider replacing 'In addition' with also given use of additionally in the preceding line.

7. Line 314 - needs a , after In Zambia

8. Line 331 - 'and onto health facilities' is awkward. Consider rephrasing.

9. Line 356 - Remove 'on' in directly on from

10. Line 363 - Consider a comma after (2015-2020)

11. Line 386-87 - use " or ' - currently mixed up. Also, ? should be within the quotation marks

12. Line 401 - consider a , before therefore.

13. Throughout numbers 1-9 should be spelled out.

14. Line 429 - Please check grammar

15. Line 446-447 - Please see repeated item API

**********

what does this mean?). If published, this will include your full peer review and any attached files.

If you choose “no”, your identity will remain anonymous but your review may still be made public.

**Do you want your identity to be public for this peer review?**  For information about this choice, including consent withdrawal, please see our Privacy Policy

Reviewer #1: No

**********

[NOTE: If reviewer comments were submitted as an attachment file, they will be attached to this email and accessible via the submission site. Please log into your account, locate the manuscript record, and check for the action link "View Attachments". If this link does not appear, there are no attachment files.]

To ensure your figures meet our technical requirements, please review our figure guidelines: https://journals.plos.org/plosone/s/figures

You may also use PLOS’s free figure tool, NAAS, to help you prepare publication quality figures: https://journals.plos.org/plosone/s/figures#loc-tools-for-figure-preparation.

NAAS will assess whether your figures meet our technical requirements by comparing each figure against our figure specifications.

# Author comment: Author response to Decision Letter 1

**DOI:** 10.1371/journal.pone.0356398.r002

2 Jul 2026

Our full and considered response to the reviewer and editor comments provided is included in our 'Response to Reviewers' cover letter.

Attachment Submitted filename: Response to Reviewers.docx

# Editor report: Decision Letter 1

**DOI:** 10.1371/journal.pone.0356398.r003

**Authors:** Sebastian Suarez Fuller

4 Aug 2026

Co-producing access to care with young people living in Zambia through digital and vending machine-enabled innovation in sexual and reproductive health: a place-based, human-centred design research approach

PONE-D-25-24514R1

Dear Dr. Darking,

We’re pleased to inform you that your manuscript has been judged scientifically suitable for publication and will be formally accepted for publication once it meets all outstanding technical requirements.

Within one week, you’ll receive an e-mail detailing the required amendments. When these have been addressed, you’ll receive a formal acceptance letter and your manuscript will be scheduled for publication.

An invoice will be generated when your article is formally accepted. Please note, if your institution has a publishing partnership with PLOS and your article meets the relevant criteria, all or part of your publication costs will be covered. Please make sure your user information is up-to-date by logging into Editorial Manager at Editorial Manager® and clicking the ‘Update My Information' link at the top of the page. For questions related to billing, please contact billing support.

If your institution or institutions have a press office, please notify them about your upcoming paper to help maximize its impact. If they’ll be preparing press materials, please inform our press team as soon as possible -- no later than 48 hours after receiving the formal acceptance. Your manuscript will remain under strict press embargo until 2 pm Eastern Time on the date of publication. For more information, please contact onepress@plos.org.

Kind regards,

Sebastian Suarez Fuller, PhD

Academic Editor

PLOS One

Additional Editor Comments (optional):

Thank you for your time and patience. Your efforts in revising this manuscript have resulted in what I believe is now a very meaningful contribution to the literature.

Reviewers' comments:

# Editor report: Acceptance letter

**DOI:** 10.1371/journal.pone.0356398.r004

**Authors:** Sebastian Suarez Fuller

PONE-D-25-24514R1

PLOS One

Dear Dr. Darking,

I'm pleased to inform you that your manuscript has been deemed suitable for publication in PLOS One. Congratulations! Your manuscript is now being handed over to our production team.

At this stage, our production department will prepare your paper for publication. This includes ensuring the following:

* All references, tables, and figures are properly cited

* All relevant supporting information is included in the manuscript submission,

* There are no issues that prevent the paper from being properly typeset

You will receive further instructions from the production team, including instructions on how to review your proof when it is ready. Please keep in mind that we are working through a large volume of accepted articles, so please give us a few days to review your paper and let you know the next and final steps.

Lastly, if your institution or institutions have a press office, please let them know about your upcoming paper now to help maximize its impact. If they'll be preparing press materials, please inform our press team within the next 48 hours. Your manuscript will remain under strict press embargo until 2 pm Eastern Time on the date of publication. For more information, please contact onepress@plos.org.

You will receive an invoice from PLOS for your publication fee after your manuscript has reached the completed accept phase. If you receive an email requesting payment before acceptance or for any other service, this may be a phishing scheme. Learn how to identify phishing emails and protect your accounts at https://explore.plos.org/phishing.

If we can help with anything else, please email us at customercare@plos.org.

Thank you for submitting your work to PLOS One and supporting open access.

Kind regards,

PLOS ONE Editorial Office Staff

on behalf of

Dr. Sebastian Suarez Fuller

Academic Editor

PLOS One
